# The Diagnostic Value of the CA19-9 and Bilirubin Ratio in Patients with Pancreatic Cancer, Distal Bile Duct Cancer and Benign Periampullary Diseases, a Novel Approach

**DOI:** 10.3390/cancers14020344

**Published:** 2022-01-11

**Authors:** Lenka N. C. Boyd, Mahsoem Ali, Laura Kam, Jisce R. Puik, Stephanie M. Fraga Rodrigues, Eline S. Zwart, Freek Daams, Barbara M. Zonderhuis, Laura L. Meijer, Tessa Y. S. Le Large, Elisa Giovannetti, Hanneke W. M. van Laarhoven, Geert Kazemier

**Affiliations:** 1 Department of Surgery, Cancer Center Amsterdam, Amsterdam UMC, VU University Medical Center (VUmc), De Boelelaan 1118, 1081 HZ, Postbus 7057, 1007 MB Amsterdam, The Netherlands; l.boyd@amsterdamumc.nl (L.N.C.B.); m5.ali@student.vu.nl (M.A.); l.kam@amsterdamumc.nl (L.K.); j.puik@amsterdamumc.nl (J.R.P.); s.m.fragarodrigues@amsterdamumc.nl (S.M.F.R.); e.zwart@amsterdamumc.nl (E.S.Z.); f.daams@amsterdamumc.nl (F.D.); bm.zonderhuis@amsterdamumc.nl (B.M.Z.); l.meijer@amsterdamumc.nl (L.L.M.); t.lelarge@amsterdamumc.nl (T.Y.S.L.L.); g.kazemier@amsterdamumc.nl (G.K.); 2 Lab of Medical Oncology, Department of Medical Oncology, Cancer Center Amsterdam, Amsterdam UMC, VU University Medical Center (VUmc), De Boelelaan 1118, 1081 HZ, Postbus 7057, 1007 MB Amsterdam, The Netherlands; h.vanlaarhoven@amsterdamumc.nl; 3 Department of Surgery, Erasmus MC-University Medical Center, Doctor Molewaterplein 40, 3015 GD Rotterdam, The Netherlands; 4 Department of Surgery, Dijklander Ziekenhuis Locatie Hoorn, Maelsonstraat 3, 1624 NP Hoorn, The Netherlands; 5 Cancer Pharmacology Lab, AIRC Start-Up Unit, Fondazione Pisana per la Scienza, Via Ferruccio Giovannini 13, San Giuliano Terme PI, 56017 Pisa, Italy; 6 Department of Medical Oncology, Cancer Center Amsterdam, Amsterdam UMC, University of Amsterdam, De Boelelaan 1118, 1081 HZ, Postbus 7057, 1007 MB Amsterdam, The Netherlands

**Keywords:** CA19-9, bilirubin, CA19-9-bilirubin-ratio, pancreatic cancer, pancreatic adenocarcinoma, biliary tract cancer, distal bile duct cancer, benign periampullary diseases, diagnostic biomarker, liquid biopsy

## Abstract

**Simple Summary:**

Distinguishing cancer in the head of the pancreas, distal bile duct cancer, and benign periampullary conditions, is complex due to often overlapping symptoms. Accurate diagnosis is important for early treatment initiation and improvement of patient prognoses. The aim of this study was to assess the diagnostic potential of the ratio of CA19-9 with bilirubin in patients with cancer of the pancreatic head, distal bile duct cancer and benign diseases of the periampullary region prior to treatment. We confirmed in a population of 232 patients with hepatopancreaticobiliary disease in the periampullary region, that the ratio of CA19-9 and bilirubin showed higher diagnostic power than analysis of CA19-9 and bilirubin levels alone and therefore could be useful to aid early diagnosis and improve treatment of patients in the future.

**Abstract:**

Distinction of pancreatic ductal adenocarcinoma (PDAC) in the head of the pancreas, distal cholangiocarcinoma (dCCA), and benign periampullary conditions, is complex as they often share similar clinical symptoms. However, these diseases require specific management strategies, urging improvement of non-invasive tools for accurate diagnosis. Recent evidence has shown that the ratio between CA19-9 and bilirubin levels supports diagnostic distinction of benign or malignant hepatopancreaticobiliary diseases. Here, we investigate the diagnostic value of this ratio in PDAC, dCCA and benign diseases of the periampullary region in a novel fashion. To address this aim, we enrolled 265 patients with hepatopancreaticobiliary diseases and constructed four logistic regression models on a subset of patients (*n* = 232) based on CA19-9, bilirubin and the ratio of both values: CA19-9/(bilirubin^−1^). Non-linearity was investigated using restricted cubic splines and a final model, the ‘Model Ratio’, based on these three variables was fitted using multivariable fractional polynomials. The performance of this model was consistently superior in terms of discrimination and calibration compared to models based on CA19-9 combined with bilirubin and CA19-9 or bilirubin alone. The ‘Model Ratio’ accurately distinguished between malignant and benign disease (AUC [95% CI], 0.91 [0.86–0.95]), PDAC and benign disease (AUC 0.91 [0.87–0.96]) and PDAC and dCCA (AUC 0.83 [0.74–0.92]) which was confirmed by internal validation using 1000 bootstrap replicates. These findings provide a foundation to improve minimally-invasive diagnostic procedures, ultimately ameliorating effective therapy for PDAC and dCCA.

## 1. Introduction

Pancreatic ductal adenocarcinoma (PDAC) is the most common malignancy that originates in the pancreas with an aggressive character and a dismal 5-year survival rate of 10.8% [1,2,3]. Due to its lethality, PDAC is expected to become the third leading cause of cancer-related deaths in Europe in 2025 [1,4]. PDAC located in the pancreatic head often has similar nonspecific clinical symptoms, histological features and lesions that are indistinguishable on imaging to distal cholangiocarcinoma, which makes it problematic to discriminate between these two tumour types [5]. Distal cholangiocarcinoma (dCCA) originates from the distal bile duct and has a slightly better, though still poor, prognosis [6]. Not only is it complex to differentiate between these tumour types, but also distinguishing these tumours from benign conditions can be difficult, due to a similarity in clinical presentation, as benign conditions can also be characterized by weight loss and jaundice [7]. Invaluable research efforts have shown that approximately 7% of patients undergoing surgery for a suspicion of PDAC were eventually found to have a benign condition for which surgery was not required [7,8]. The difference in therapeutic approach of PDAC and dCCA, plus the importance of preventing unnecessary surgery in the case of benign disease, underlines the necessity of correct pre-treatment diagnosis.

The current diagnostic process for these diseases is based on clinical symptoms of the patient, radiological findings, pathological confirmation of the diagnosis by fine-needle aspiration or brush cytology, and measurement of the clinical disease marker carbohydrate antigen 19-9 (CA19-9). Endoscopic ultrasound-guided fine-needle aspiration (EUS FNA), fine needle biopsy (FNB) or brush cytology are invasive techniques, and despite the positively high accuracy of EUS-guided FNA or FNB [9] these techniques are frequently unable to adequately differentiate PDAC from dCCA, and the precise diagnosis, stage and prognosis often only becomes evident after surgical resection [10]. Even then, confirmation of an accurate diagnosis can be challenging due to the high stromal density of these tumours [2].

Of the aforementioned standard biomarkers, CA19-9 is the most commonly used non-invasive and best validated serum tumour marker for PDAC in symptomatic patients [11]. Recent studies also show a strong correlation of CA19-9 in bile duct cancer and have suggested it serves as a prognostic biomarker in this tumour type [12]. Normally synthesized by normal human pancreatic and biliary ductal cells, CA19-9 is present in small amounts in serum but can also be over expressed in several benign gastrointestinal disorders [11]. Elevated levels of CA19-9 are often found in patients suffering from PDAC or dCCA, as well as in patients with benign disease with obstructive jaundice [6]. Besides this, false negative results are found in subjects with Lewis (a-b) genotype, as these patients are unable to synthesize CA19-9 [13]. All these critical aspects hamper the diagnostic yield of CA19-9. Sensitivity and specificity of CA19-9 in diagnosing cancers arising from the biliary tree are reported at around 77–78% and 81–84%, respectively [10], and for PDAC, between 79% and 95%, and between 82% and 91%, respectively [14,15,16,17]. Consequently, CA19-9 is mainly used in monitoring conditions and follow-up after therapy [18].

In recent years, alongside an increase in advancements of technology and techniques, a vast amount of research has been done to identify novel non-invasive diagnostic biomarkers to improve diagnosis and consequently to improve treatment of PDAC. Indeed, several research groups are evaluating novel potential diagnostic proteins, circulating tumour DNA, circulating tumour cells and DNA, methylation, RNA of tumour-educated platelets, or microRNAs [19,20,21,22]. These studies have shown promising results, however, none of these metrics have reached the phase of clinical validation to pursue implementation in the clinic [23]. As a result, CA19-9 remains the only clinically validated blood biomarker for these tumours. Thus, it is crucial to recognize the potential of this commonly used ‘standard’ biomarker and to implement it accordingly for maximum diagnostic proficiency. The combination of known clinical variables and novel tumour biomarkers can indeed be the key to a more enhanced and accurate diagnostic process.

To improve the diagnostic accuracy of CA19-9, research suggests that adjusting CA19-9 levels to serum bilirubin levels can improve the process of differentiating between benign and malignant hepatopancreaticobiliary diseases [24,25]. Under normal conditions, most of the bilirubin in the human body (80%) originates from haemoglobin of senescent erythrocytes [26]. An obstruction in the biliary tree caused by a benign process can cause bilirubin levels to rise, though bilirubin reaches significantly higher levels when the obstruction is caused by malignant disease [27]. The precise relationship between CA19-9 and bilirubin is not fully understood yet, but research evidence suggests that CA19-9 is elevated in patients with hyperbilirubinemia, resulting in a reduced specificity of CA19-9 in diagnosing hepatopancreaticobiliary disease [28]. The pathophysiological explanation for this could be that the biliary tract functions as an excretion pathway for CA19-9 to the liver and, therefore, an obstruction causing hyperbilirubinemia could block the bile flow, resulting in higher serum levels of CA19-9 [28]. In the light of this hypothesis, studies suggest that adjusting CA19-9 to bilirubin by calculating the CA19-9/bilirubin ratio could have a stronger diagnostic value than CA19-9 alone [29,30].

However, the CA19-9/bilirubin ratio in which CA19-9 is divided by bilirubin in regression analysis has been questioned by statisticians [31,32], as it implies a linear relationship between these markers and does not accurately reflect a patient’s clinical status, i.e., a patient with a high level of CA19-9 and a high level of bilirubin could have a similar ratio to a patient with a low level of CA19-9 and bilirubin. Considering this, it is suggested to evaluate ratios as the value of dividing CA19-9 by the reciprocal of bilirubin (e.g., CA19-9/(bilirubin^−1^)), based on the mathematical equation of Kronmal and collaborators, which does take this consideration into account [32].

Therefore, in this study, we aimed to assess the diagnostic value of CA19-9 and its ratio with the reciprocal of serum bilirubin in pre-treatment patients with PDAC, dCCA and benign periampullary diseases to benefit future clinical implementation of these markers for enhancement of diagnostic accuracy.

## 2. Materials and Methods

### 2.1. Study Population and Design

The study design and protocol were approved by the local Medical Ethics Board of the Amsterdam UMC, VU University Amsterdam (#2016.510) in accordance with the ethical guidelines of the Declaration of Helsinki. Before study participation, written informed consent was obtained from all 265 participants. Patients with hepatopancreaticobiliary diseases that visited the hepatopancreaticobiliary clinic between 2015 and 2020 for various indications were included in the study. Final diagnosis was based on histopathological confirmation, or examination of the resected specimen by the pathologist in the case that surgery was performed. Clinicopathological characteristics were collected in a prospectively maintained database. The clinically relevant variables that were extracted included gender, age, date of diagnosis, date of death, tumour characteristics, tumour stage, serum CA19-9 and serum bilirubin levels. All patients with benign diseases that did not originate from the periampullary region, or are part of a systemic immune disease, were excluded from the analyses. Table 1 provides a complete overview of inclusion and exclusion criteria.

### 2.2. Detection of CA19-9 and Bilirubin Levels

CA19-9 and bilirubin levels were determined in the blood of patients prior to treatment and without any medical intervention at the Clinical Chemistry Laboratory, Amsterdam UMC location VUMC (Amsterdam, The Netherlands). The CA19-9 expression levels were determined by the Immunometric assay, Luminescence (Advia Centaur XP, Siemens Healthineers, Malvern, PA, USA) and bilirubin levels by the colorimetric diazo method (Bilirubin Total Gen.3, Roche Diagnostics International, Rotkreuz, Switzerland). The CA19-9 upper limit of normal (ULN) was set at 37 U/mL and levels were classified as high or low based on mean values. A bilirubin level of ≥20 μmol/L was considered an elevated level of bilirubin. The CA19-9 to bilirubin ratio was calculated by the formula CA19-9/(bilirubin^−1^). This ratio was calculated only in the case that both values were obtained on the same day of blood withdrawal.

### 2.3. Statistical Analysis

Normality of distributions of the patient baseline characteristics was assessed with the D’Agostino-Pearson omnibus test. If distributions were normal, analysis of variance tests were used for multiple-group comparisons. If distributions were not normally distributed, the Kruskal–Wallis test was used for comparisons between multiple groups, followed by Dunn’s post-hoc test. Pearson’s chi-squared test was used for comparing categorical variables across multiple groups.

For the prediction models, logistic regression models were constructed and based on CA19-9, bilirubin and the ratio between CA19-9 and bilirubin: CA19-9/(bilirubin^−1^). Nonlinear relationships between continuous predictors and binary outcomes (e.g., PDAC and dCCA vs. benign, PDAC vs. benign disorders and PDAC vs. dCCA) were investigated using restricted cubic splines (RCS) and multivariable fractional polynomials (MFP). A total of four logistic regression models were constructed: a robust model with transformations of CA19-9, bilirubin and (CA19-9/(bilirubin^−1^)) using MFPs (‘Model Ratio’); a model based on CA19-9 plus bilirubin without consideration of the ratio; a model based on CA19-9 only; and a model based on bilirubin only. Discrimination and calibration were assessed for each model and each comparison. The discriminatory value of the model was defined as AUC (95% CI), with an ‘optimal’ cut-off based on the Youden index with associated sensitivity (SEN) and specificity (SPE) and 95% CI. Model calibration was assessed with the integrated calibration index (ICI) and with overfitting-corrected calibration curves based on loess smoothing and 1000 bootstrap replicates [33]. Decision curve analysis, based on 1000 repeats of 5-fold cross-validation, was carried out to evaluate the diagnostic net benefit of the model [34].

Internal validation was performed using 1000 bootstrap replications to estimate over-optimism of the models and penalized maximum likelihood estimation was subsequently used to adjust the Model Ratio for overoptimism. The results of this penalized model, as well as for the three models based only on CA19-9, bilirubin and the combination of CA19-9 and bilirubin are reported in Supplementary Table S1. All analyses were performed in R, version 4.1.0 (R Foundation for Statistical Computing, Vienna, Austria) using the Hmisc, mfp, pROC, rmda and rms packages [34,35].

## 3. Results

### 3.1. Patient Characteristics

A total of 265 patients with hepatopancreaticobiliary diseases were included in the study, of which the baseline characteristics are summarized in Table 2.

The mean age of the patients in this total cohort of 265 patients was between 65 and 69 years, and 145 patients were male (54.7%). A total of 212 patients were diagnosed with malignant tumours and 53 with benign diseases of the periampullary region, i.e., either diagnosed with cholangitis, chronic pancreatitis, intraductal papillary mucinous neoplasm (IPMN), pancreatic lipomas, pancreatic cysts, or benign cystic bile duct stenosis. Of the patients diagnosed with malignant tumours, 178 were diagnosed with PDAC and 34 with dCCA.

**Figure 1:**
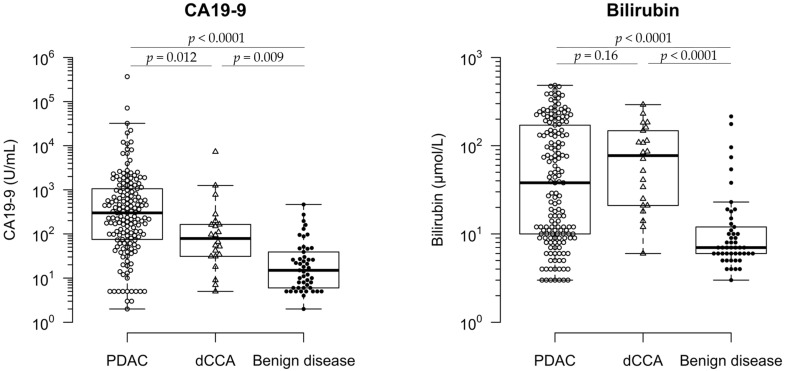
Evaluation of baseline CA19-9 and bilirubin levels of all patients. Box-and-whisker plots of CA19-9 and bilirubin levels in patients with PDAC = pancreatic ductal adenocarcinoma, dCCA = distal cholangiocarcinoma, and benign disease. The median is shown by a thick stripe, and box depicts the interquartile range (IQR) with 75th percentile + 1.5*IQR and 25th percentile-1.5*IQR (whiskers). Significant comparisons are shown with *p* values described below. *p*-values were calculated with the Kruskal–Wallis test followed by Dunn’s test for multiple comparisons. The false discovery rate was controlled with the Benjamini–Hochberg procedure.

Baseline CA19-9 levels were analysed in the total patient cohort of 265 patients. Baseline CA19-9 levels varied between the groups, with a higher average of CA19-9 levels in the malignant groups compared to the benign group. The interpretation of this relates to the fact that CA19-9 has been described to be elevated in benign conditions mostly due to biliary obstruction, whereas it has been hypothesized that CA19-9 is actually secreted by tumours and thus causes higher concentrations of this marker [29]. Figure 1 shows two whisker boxplots comparing median CA19-9 and bilirubin levels within the malignant groups and benign group.

### 3.2. Characteristics of the Subset of Patients for Calculation of the CA19-9 and Bilirubin Ratio

Of the total cohort, a subset of 232 patients was analysed for calculation of the CA19-9 and bilirubin ratio. Table 3 shows the baseline characteristics of this subset which was further analysed for calculation of the CA19-9 and bilirubin ratio. In this subset, the mean age of the patients in all the groups were between 66 and 69 years, and 126 patients were male (54.3%). A total of 183 patients were diagnosed with malignant tumours and 49 with benign diseases of the periampullary region. Of the patients diagnosed with malignant tumours, 161 were diagnosed with PDAC and 22 with dCCA. In the group of patients with PDAC, 102 patients (63.4%) were diagnosed with stage 1–2 disease and 59 patients (36.6%) with stage 3–4 disease. In the group of patients with dCCA, 14 patients (63.6%) were diagnosed with stage 1–2 disease and 8 patients (36.4%) with stage 3–4 disease.

### 3.3. Prediction Models

**Figure 2:**
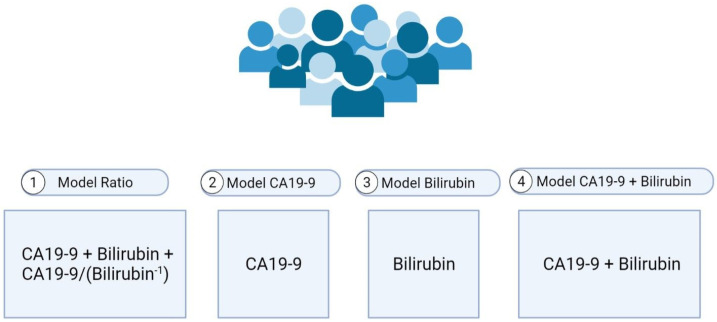
Four logistic regression models adopted in this study.

To investigate the diagnostic value of CA19-9 and bilirubin in the cohort of 232 patients with histologically confirmed hepatopancreaticobiliary disease, four logistic regression models based on CA19-9, bilirubin, and CA19-9/(bilirubin^−1^) were constructed which are graphically shown below in Figure 2. The first model, ‘Model Ratio’, was based on the variables CA19-9 and bilirubin and the ratio CA19-9/(bilirubin^−1^). The second model, called ‘Model CA19-9′, was based on levels of CA19-9; the third model ‘Model Bilirubin’ was based on levels of bilirubin, and the fourth model ‘Model CA19-9 + Bilirubin’ consisted of the values of CA19-9 + bilirubin without taking the ratio into consideration.

### 3.4. Comparisons of the Prediction Models

Subsequently, AUC, SEN, SPEC and the accuracy of these models were compared. The ‘Model Ratio’ showed the highest AUCs with 95% confidence interval (CI) in discriminating between PDAC and dCCA vs. benign (AUC = 0.91 [0.86–0.95]), PDAC vs. benign (AUC = 0.91 [0.87–0.96]), and PDAC vs. dCCA (AUC = 0.83 [0.74–0.92]), compared to the models that included only the individual markers, or the markers taken together without consideration of the ratio. A full overview of the comparisons of AUC, SEN, SPE and accuracy is reported in Table 4.

**Figure 3:**
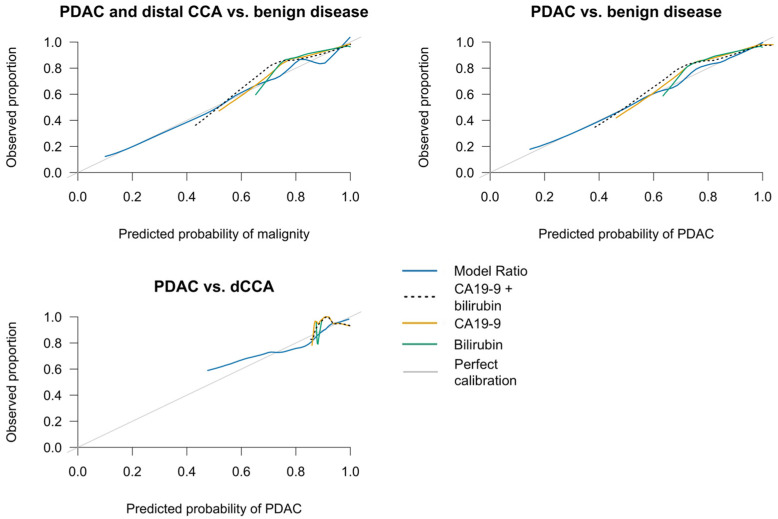
Overfitting-corrected calibration curve using a loess nonparametric smoother and 1000 bootstrap repetitions.

To assess model calibration, overfitting-corrected calibration curves were constructed based on loess smoothing and 1000 bootstrap replicates. In Table 4, the Integrated Calibration Index (ICI) shows the weighted average of the difference between the model’s predicted probability and the observed probability of having a specific diagnosis. The Model Ratio was consistently the best calibrated model, as it predicted the probability of having a specific diagnosis more accurately than the other models. The Model Ratio showed the lowest ICI for predicting malign vs. benign disease (0.029), PDAC versus benign disease (0.019) and PDAC versus dCCA (0.027). Figure 3 supports this notion as it depicts the calibration curves for the predicted probabilities. The predicted probabilities when using the ‘Model Ratio’ correspond most with the observed probability of disease.

### 3.5. Model Ratio ROC Curves

**Figure 4:**
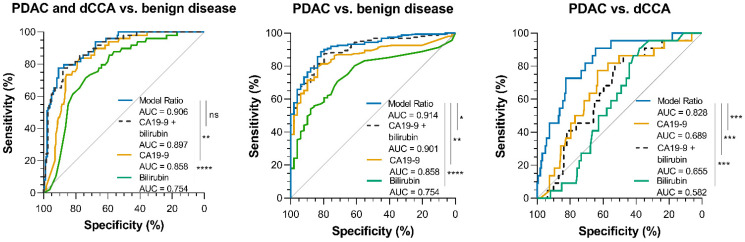
ROC curves and AUC comparisons of the ‘Model Ratio’ with the models using only CA19-9 and bilirubin. Malign vs. benign; Model ratio vs. CA19-9: *p* = 0.002 (**: *p* < 0.01), Model ratio vs. bilirubin: *p* = 3.0 × 10^−7^ (****: *p* < 0.0001) Model ratio vs. CA19-9 + bilirubin: *p* = 0.071 (ns: *p* > 0.05) PDAC vs. benign; Model ratio vs. CA19-9: *p* = 0.0011 (**: *p* < 0.01), Model ratio vs. bilirubin: *p* = 3.3 × 10^−7^ (****: *p* < 0.0001); Model ratio vs. CA19-9 + bilirubin: *p* = 0.048 (*: *p* < 0.05) PDAC vs. dCCA; Model ratio vs. CA19-9: *p* = 0.0009 (***: *p* < 0.001) Model ratio vs. bilirubin: *p* = 0.0002 (***: *p* < 0.001) Model ratio vs. CA19-9 + bilirubin: *p* = 0.0004 (***: *p* < 0.001).

The performance of the ‘Model Ratio’ was consistently superior in terms of discrimination and calibration, compared to the models based on CA19-9 or bilirubin alone, and similar, or superior to, the models based on CA19-9 plus bilirubin. Figure 4 shows the ROC curves and the comparisons of the AUC values for malign versus benign, PDAC versus benign and PDAC versus dCCA.

The ‘Model Ratio’ accurately distinguished between malign and benign disease (AUC [95% CI], 0.91 [0.86–0.95]), PDAC and benign disease (AUC 0.91 [0.87–0.96]) and PDAC and dCCA (AUC 0.83 [0.74–0.92]) as indicated by the blue line in Figure 4, which shows superiority to the AUC’s of the models using CA19-9 plus bilirubin, or CA19-9 and bilirubin alone (in the malign versus benign group AUC’s of 0.897, 0.848 and 0.770 respectively, PDAC versus benign group AUC’s of 0.901, 0.858 and 0.754 respectively, and in the PDAC versus dCCA group AUC’s of 0.655, 0.689 and 0.582, respectively).

### 3.6. Standardized Net Benefit Analysis

**Figure 5:**
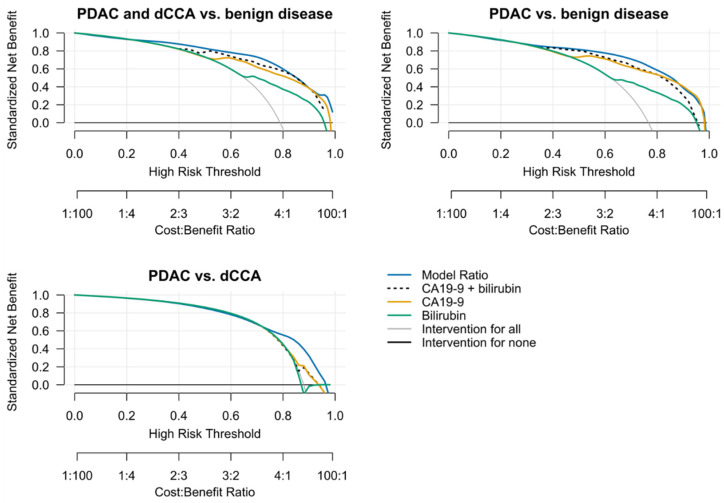
Overoptimism-corrected decision curve analysis using 1000 repeats of 5-fold cross-validation. Five-fold cross-validation was repeated 1000 times and results were averaged across all repetitions to obtain robust estimates of the standardized net benefit at each threshold. Briefly, the clinical usefulness of a diagnostic test is assessed by comparing standardized net benefit over a range of probability thresholds. Net benefit is defined as the number of true positives found at a certain cut-off penalized by the number of false positives found for that cut-off, where the weight of the penalty is defined by the relative importance of finding a false positive. For further information regarding decision curve analysis, the reader is referred to [33,34].

To investigate the clinical usefulness of the models, decision curve analysis was carried out and the net benefit of the models was compared to an ‘intervention for all’ and ‘intervention for none’ strategy. This analysis indicated that the net benefit of the ‘Model Ratio’ was either superior or equal to the net benefit of the other models across all risk thresholds (Figure 5).

Internal validation was performed using 1000 bootstrap replicates to determine overoptimism-corrected performance estimates. The ‘Model Ratio’ proved superior after correction for overoptimism of the performance estimates with superior AUC’s and calibration compared to the other models using CA19-9 plus bilirubin or CA19-9 or bilirubin alone (Supplementary Table S1).

## 4. Discussion

In this study, we show that the model based on the values of CA19-9 and bilirubin and the ratio CA19-9/(bilirubin^−1^), shows increased sensitivity, specificity, and higher AUC values than the prediction models using CA19-9 and bilirubin levels alone, and can accurately differentiate between patients with PDAC, dCCA or benign disease. This presents a novel way to use the known and clinically available biomarkers CA19-9 and bilirubin to benefit diagnostic accuracy. Importantly, this study demonstrates that the diagnostic value of the CA19-9 and bilirubin ratio is also relevant to cholangiocarcinomas, which has not been described in research efforts before. The poor prognosis of PDAC and dCCA is partially determined by the lack of possibilities for early diagnosis and the lack of disease-specific biomarkers [36]. Levels of CA19-9 and bilirubin, which are commonly measured in the clinic, play a dynamic part of hepatopancreaticobiliary disease and thus in PDAC and dCCA pathophysiology. For this reason, a better understanding of these markers and their proportionality is crucial for advancement of the current diagnostic process and ultimately for improvement of therapeutic strategies and prognoses for patients with PDAC and dCCA.

It is of utmost importance to carry out appropriate, clinically useful, and statistically sound subgroup comparisons, which enable researchers to evaluate the performance of biomarkers and models in the clinical setting. Hence, one of the strengths of the ‘Model Ratio’ is that it is able to detect PDAC with an AUC of 0.91 and can distinguish it from the control group consisting of patients with benign diseases, of which in both groups elevated, low or normal CA19-9 and bilirubin levels can be present. The ‘Model Ratio’ proved superior during internal validation (based on 1000 bootstrap replicates), which is crucial regarding the fact that models will attempt to fit the used dataset as optimally as possible, causing the problem that the model is very specific to the current dataset and cannot be generalized to larger populations. As a result, it is possible that the AUC fails during internal and external validation, which was limited in this study and even after correction for overfitting, the ‘Model Ratio’ outperformed the other models. However, even though an extensive internal validation was performed showing positively high AUC’s, the lack of an external validation cohort remains a limitation of this study. As such, efforts towards a large external validation cohort are currently being made on a national level to further assess the clinical utility of the CA19-9 and bilirubin ratio for patients suffering from PDAC and dCCA.

In this ongoing and future research, other limitations of using bilirubin or CA19-9 as a diagnostic tool should be further investigated, for instance, by additionally focusing on cohorts of subjects that suffer from comorbidity that simultaneously influences bilirubin or CA19-9 expression (colon cancer, gastric cancer). Besides this, it would be of value to evaluate bilirubin and CA19-9 levels after stent placement, as hypothetically in the case that the correlation of the two biomarkers remains stable regardless of stent placement, this would provide additional reinforcement of the added benefit of the CA19-9/bilirubin ratio in the diagnosis of PDAC. Furthermore, extra attention should be drawn to the patients with IgG4 disease, to adequately assess the predictive performance and clinical utility of the model incorporating IgG4 levels, as well as IPMN patients, as caution is advised when subjects develop into a pre-malignant stage in which both the diagnosis benign/malignant as well as the ratio of bilirubin and CA19-9 is more ambivalent. Including patients with IgG4-mediated disease would entail extending the ‘Model Ratio’ to include serum IgG4, which requires additional predictor parameters to be estimated and thus increases the necessary minimum sample size, especially if non-linear terms are modelled. Importantly, these levels should be measured for comparison in all patients, which is not standard practice in the clinic. As enough patients with IgG4-mediated disease and IgG4 levels in the total cohort are not currently available in our dataset, this remains a limitation of our study. Like IgG4-mediated disease, additional clinical diagnoses are needed for validation studies, including biliary stenosis without a mass, as these diagnoses are often challenging. Additionally, an interesting avenue of approach for follow-up studies would be to evaluate the prognostic potential of the ratio and whether the ‘Model Ratio’ is able to accurately stratify patients into high- and low-risk categories.

Determining a clinical diagnosis based on a prediction model remains a complex and much debated issue, and the use of AUCs and their additive value in the clinic is frequently questioned, as they are “statistical abstractions not directly informative about clinical value” [37,38,39]. However, these prediction models are ultimately essential for estimations of the physician and when constructed and evaluated correctly by investigating non-linear relationships and assessing discrimination, calibration and clinical utility, these models can be useful and crucial tools for clinical decision making [37,40]. Besides this, in the context of cost effectiveness analyses, which depend on discriminatory values as AUC, SEN and SPEC, these prediction models are indispensable. Two fairly recent publications which discuss the cost effectiveness of PDAC surveillance state that a biomarker with 96% specificity and 65% sensitivity could already result in an acceptable cost-benefit analysis [41] and that screening of high-risk pancreatic cancer patients could result in an increase of survival by 30–40% and is cost-effective when a test shows a sensitivity above 88% and specificity of at least 85% [42]. Our ‘Model Ratio’ fulfils these criteria with a SEN of 65.0, at a SPE of 96 (95% CI 87.8–100.0) [95% CI, 10,000 bootstrap replicates] and a SEN of 89, with a SPE of 82 (95% CI 87.8–100.0) [95% CI, 10,000 bootstrap replicates]. In addition, no extra or novel analyses are needed to apply the ‘Model Ratio’ into clinical care, as both CA19-9 and bilirubin values are a common part of the current standardized practice and little adjustments are needed to calculate the ratio. The ongoing nationwide, large-scale validation, including cost-benefit analyses, will facilitate further insight in the most optimal cut-off and help facilitate clinical use.

Our findings should prompt further studies to standardize implementation of the ratio of CA19-9/(bilirubin^−1^) in the standard laboratory collection protocol. Additionally, it would be important to further investigate the additive value of other standard clinically measured variables like creatinine, haemoglobin, albumin, and measurements of liver enzymes that have been described to vary in different hepatopancreaticobiliary patient groups [43], and also in combination with novel tumour specific blood-based biomarkers. However, it remains to be investigated how feasible it is to combine various standard clinical markers and novel blood-based biomarkers in a test which is easy to implement and practical, and which is equally accurate or superior to invasive histologically confirmed fine needle aspiration or biopsy. Our aim is not to specifically provide an alternative to these techniques, but to support them and where possible to protect a patient from an invasive procedure and to diagnose as quickly and efficiently as possible.

Therefore, further clinically oriented research is necessary to validate the ‘Model Ratio’ and to investigate the possibility of combining various novel diagnostic biomarkers and the ratio of CA19-9 and bilirubin levels.

## 5. Conclusions

We show an improved diagnostic potential of the current clinically employed biomarker CA19-9 as a ratio with bilirubin for diagnosis of PDAC and dCCA. Our study demonstrates that the ratio of CA19-9 and bilirubin can distinguish PDAC from dCCA and from benign diseases highly accurately and thus can benefit diagnosis. These novel insights on the CA19-9 and bilirubin ratio highlight useful repurposing of known biomarkers for the future improvement of minimally invasive diagnostic procedures and ultimately an improvement of treatment for patients suffering from PDAC and dCCA.

## Figures and Tables

**Table 1:** Inclusion and exclusion criteria.

Inclusion Criteria	Exclusion Criteria
Patients ≥ 18 year Informed consent	Patients < 18 year No consent
Included tumours: Distal cholangiocarcinoma (dCCA) Pancreatic ductal adenocarcinoma (PDAC) Histopathologically confirmed	Excluded tumours: Hepatocellular carcinoma (HCC) Gallbladder cancer Metastases from other primary malignancies Not histopathologically confirmed
Included benign diseases: Cholangitis on basis of choledocholithiasis Chronic pancreatitis Intraductal Papillary Mucinous Neoplasm (IPMN) Pancreatic lipomas Pancreatic cysts Benign CBD stenosis	Excluded benign diseases: Primary Sclerosing Cholangitis (PSC) Hepatitis Liver cirrhosis Neuroendocrine tumours Cholecystitis IgG4 autoimmune pancreatitis

**Table 2:** Baseline Characteristics.

Characteristic	PDAC (*n* = 178)	Distal CCA (*n* = 34)	Benign Disease (*n* = 53)	*p* Value
Age, mean (SD)—yr	68.1 (9.5)	69.4 (10.0)	65.3 (12.4)	0.233
Sex—*n* (%)				0.617
Female	84 (47.2)	15 (44.1)	21 (39.6)	
Male	94 (52.8)	19 (55.9)	32 (60.4)	
Tumor stage—*n* (%)				0.956
Stage I-II	116 (65.2)	22 (64.7)	0 (0)	
Stage III-IV	62 (34.8)	12 (35.3)	0 (0)	
N/A	0 (0)	0 (0)	53 (100)	
CA19-9, median (IQR) - U/mL	243 (72–963)	104 (43–292)	15 (6–39)	<0.0001
CA19-9—*n* (%)				<0.0001
Normal	29 (16.3)	27 (79.4)	39 (73.6)	
Elevated	149 (83.7)	7 (20.6)	14 (26.4)	
Bilirubin, median (IQR) - μmol/L	38 (10–156)	85 (27–169)	7 (6–13)	<0.0001
Bilirubin—*n* (%)				<0.0001
Normal	79 (44.4)	5 (14.7)	45 (84.9)	
Elevated	99 (55.6)	29 (85.3)	8 (15.1)	

AJCC Cancer Staging Manual, 8th Edition. PDAC = pancreatic ductal adenocarcinoma, Distal CCA = distal cholangiocarcinoma, CA19-9 = carbohydrate antigen 19-9; n = number of patients; SD = standard deviation, IQR = interquartile range.

**Table 3:** Baseline Characteristics of the Included Patients for Calculation of the CA19-9 and Bilirubin Ratio.

Characteristic	PDAC (*n* = 161)	Distal CCA (*n* = 22)	Benign Disease (*n* = 49)	*p* Value
Age, mean (SD)—yr	68.8 (9.1)	69.9 (10.3)	66.3 (12.1)	0.384
Sex—*n* (%)				0.283
Female	79 (49.1)	9 (40.9)	18 (36.7)	
Male	82 (50.9)	13 (59.1)	31 (63.3)	
Tumor stage—*n* (%)				0.979
Stage I-II	102 (63.4)	14 (63.6)	0 (0)	
Stage III-IV	59 (36.6)	8 (36.4)	0 (0)	
N/A	0 (0)	0 (0)	49 (100)	
CA19-9, median (IQR) - U/mL	300 (75–1059)	80 (32–164)	15 (6–39)	<0.0001
CA19-9—*n* (%)				<0.0001
Normal	24 (14.9)	15 (68.2)	36 (73.5)	
Elevated	137 (85.1)	7 (31.8)	13 (26.5)	
Bilirubin, median (IQR) - μmol/L	38 (10–171)	78 (22–140)	7 (6–12)	<0.0001
Bilirubin—*n* (%)				<0.0001
Normal	71 (44.1)	4 (18.2)	42 (85.7)	
Elevated	90 (55.9)	18 (81.8)	7 (14.3)	

AJCC Cancer Staging Manual, 8th Edition. Distal CCA = distal cholangiocarcinoma, PDAC = pancreatic ductal adenocarcinoma, CA19-9 = carbohydrate antigen 19-9; n = number of patients; SD = standard deviation, IQR = interquartile range (25th–75th percentile).

**Table 4:** Comparisons of the four models in the malign vs. benign, PDAC vs. benign and PDAC vs. dCCA group.

Comparison	Model Ratio	Model CA19-9	Model Bilirubin	Model CA19-9 + Bilirubin
PDAC and dCCA vs. benign disease**—***n* = 183 vs. 49				
AUC (95% CI)	0.906 (0.863–0.949)	0.849 (0.796–0.902)	0.770 (0.703–0.837)	0.897 (0.852–0.943)
Cut-off	0.712	37 U/mL	20 μmol/L	0.508
SEN (95% CI)	90.0	83.1 (77.6–88.5)	59.0 (51.9–66.1)	90.0
SPE (95% CI)	77.6 (65.3–87.8)	73.5 (61.2–85.7)	85.7 (75.5–93.9)	65.3 (53.1–77.6)
Accuracy (95% CI)	87.4 (83.2–91.4)	81.0 (75.9–86.2)	64.7 (58.6–70.3)	84.1 (79.3–88.4)
ICI (95% CI)	0.029 (0.014–0.061)	0.047 (0.014–0.088)	0.048 (0.015–0.088)	0.060 (0.028–0.091)
PDAC vs. benign disease**—***n* = 161 vs. 49				
AUC (95% CI)	0.914 (0.874–0.955)	0.858 (0.806–0.910)	0.754 (0.684–0.824)	0.901 (0.856–0.945)
Cut-off	0.629	37 U/mL	20 μmol/L	0.455
SEN (95% CI)	90.0	85.1 (79.5–90.1)	55.9 (48.5–63.4)	90.0
SPE (95% CI)	80.0 (67.4–89.8)	73.5 (61.2–85.7)	85.7 (75.5–93.9)	65.3 (51.0–77.6)
Accuracy (95% CI)	87.1 (82.4–91.4)	82.4 (77.1–87.6)	62.9 (56.7–69.1)	84.3 (79.5–89.1)
ICI (95% CI)	0.019 (0.016–0.069)	0.046 (0.022–0.094)	0.045 (0.024–0.098)	0.053 (0.029–0.094)
PDAC vs. distal CCA**—***n* = 161 vs. 22				
AUC (95% CI)	0.828 (0.740–0.915)	0.689 (0.581–0.796)	0.582 (0.486–0.678)	0.655 (0.549–0.761)
Cut-off	0.900	37 U/mL	20 μmol/L	0.868
SEN (95% CI)	64.6 (57.1–72.1)	85.1 (79.5–90.1)	44.1 (36.0–51.6)	52.8 (45.3–60.3)
SPE (95% CI)	90.9 (77.3–100.0)	31.8 (13.6–50.0)	81.8 (63.6–95.5)	81.8 (63.6–95.5)
Accuracy (95% CI)	67.8 (61.2–74.3)	78.7 (73.2–84.2)	48.6 (41.5–55.2)	56.3 (49.2–63.4)
ICI (95% CI)	0.027 (0.014–0.060)	0.062 (0.016–0.109)	0.061 (0.029–0.104)	0.044 (0.012–0.097)

Comparisons of the four logistic regression models. AUC = area under the curve, SEN = sensitivity, SPE = specificity, ICI = integrated calibration index, Models based on (as described above of in the methods). A cut-off of 37 U/mL for CA19-9 and 20 μmol/L for bilirubin was used. For the ‘Model Ratio’ and the ‘Model CA19-9 + bilirubin’, specificity and accuracy were calculated at a fixed sensitivity of 90% for the comparison between PDAC and dCCA vs. benign disease, and PDAC vs. benign disease. For the comparison between PDAC and dCCA, an optimal cut-off was based on the Youden index.

## Data Availability

The data presented in this study are available on request from the corresponding author.

## Supplementary Materials

The following supporting information can be downloaded at: https://www.mdpi.com/article/10.3390/cancers14020344/s1, Table S1: Internal validation with 1000 bootstrap replicates.

## Abbreviations

PDAC	Pancreatic ductal adenocarcinoma
dCCA	Distal cholangiocarcinoma
AUC	Area Under the Curve
ctDNA	Circulating tumour DNA
TEPs	Tumour-educated platelets
miRNAs	MicroRNAs
ULN	Upper limit of normal
IPMN	Intraductal papillary mucinous neoplasm
RCS	Restricted cubic splines
MFP	Multivariable fractional polynomials
SEN	Sensitivity
SPE	Specificity
PPV	Positive predictive value
NPV	Negative predictive value
