# Supplementary material for: The Diagnostic Value of the CA19-9 and Bilirubin Ratio in Patients with Pancreatic Cancer, Distal Bile Duct Cancer and Benign Periampullary Diseases, a Novel Approach
Source: Cancers (Basel). 2022 Jan 11;14(2):344. doi: 10.3390/cancers14020344 (PMC8774022; doi:10.3390/cancers14020344)
Supplement: Supplementary file 1 [file cancers-14-00344-s001.zip › cancers-1474986-supplementary.pdf]

Supplementary Materials

Table S1. Internal validation with 1000 bootstrap replicates.

Original (Corrected) Performance Measures	Model Ratio		CA19-9		Bilirubin		CA19-9 + Bilirubin	
**PDAC vs. benign**	*n* = 161 vs. 49							
AUC	0.914	(0.908)	0.858	(0.865)	0.754	(0.753)	0.901	(0.902)
$R^2_{Nagelkerke}$	0.562	(0.546)	0.421	(0.429)	0.196	(0.185)	0.490	(0.483)
Intercept	0	(−0.005)	0	(−0.074)	0	(−0.021)	0	(−0.019)
Slope	1.00	(0.989)	1.00	(1.100)	1.00	(0.996)	1.00	(0.974)
Harrell's E_{max}	0	(0.004)	0	(0.033)	0	(0.005)	0	(0.009)
**Malign vs. benign**	*n* = 222 vs. 49							
AUC	0.902	(0.898)	0.846	(0.856)	0.764	(0.764)	0.891	(0.895)
$R^2_{Nagelkerke}$	0.492	(0.470)	0.342	(0.357)	0.182	(0.174)	0.419	(0.419)
Intercept	0	(0.018)	0	(−0.166)	0	(−0.027)	0	(−0.037)
Slope	1.00	(0.976)	1.00	(1.173)	1.00	(1.010)	1.00	(1.024)
Harrell's E_{max}	0	(0.008)	0	(0.061)	0	(0.008)	0	(0.028)
**PDAC vs. dCCA**	*n* = 161 vs. 22							
AUC	0.816	(0.784)	0.689	(0.689)	0.582	(0.548)	0.655	(0.627)
$R^2_{Nagelkerke}$	0.242	(0.195)	0.030	(0.00)	0.0007	(0.00)	0.030	(0.00)
Intercept	0	(0.116)	0	(−1.555)	0	(−2.597)	0	(0.243)
Slope	1.00	(0.907)	1.00	(1.813)	1.00	(2.463)	1.00	(0.869)
Harrell's E_{max}	0	(0.043)	0	(0.326)	0	(0.441)	0	(0.080)
